# Recent advances in signal integration mechanisms in the unfolded protein response

**DOI:** 10.12688/f1000research.19848.1

**Published:** 2019-11-01

**Authors:** G. Elif Karagöz, Tomás Aragón, Diego Acosta-Alvear

**Affiliations:** 1 Max Perutz Labs Vienna, Medical University of Vienna, Vienna, Austria; 2 Department of Gene Therapy and Regulation of Gene Expression, University of Navarra, Pamplona, Spain; 3 Department of Molecular, Cellular, and Developmental Biology, University of California, Santa Barbara, Santa Barbara, Santa Barbara, CA, USA

**Keywords:** ER stress, unfolded protein response, signal transduction pathway interconnectivity

## Abstract

Since its discovery more than 25 years ago, great progress has been made in our understanding of the unfolded protein response (UPR), a homeostatic mechanism that adjusts endoplasmic reticulum (ER) function to satisfy the physiological demands of the cell. However, if ER homeostasis is unattainable, the UPR switches to drive cell death to remove defective cells in an effort to protect the health of the organism. This functional dichotomy places the UPR at the crossroads of the adaptation versus apoptosis decision. Here, we focus on new developments in UPR signaling mechanisms, in the interconnectivity among the signaling pathways that make up the UPR in higher eukaryotes, and in the coordination between the UPR and other fundamental cellular processes.

## Introduction

The unfolded protein response (UPR) comprises a collection of evolutionarily conserved signaling pathways in eukaryotes that monitor endoplasmic reticulum (ER) functions. In its most fundamental form, the UPR maintains the health of secreted and transmembrane proteins. Most of these proteins, which comprise roughly one third of the proteome, translocate into the ER for their folding and maturation. Because these proteins establish communication networks among cells and between cells and the extracellular environment, the UPR is essential for normal cell and organismal physiology. Indeed, the UPR has long been known to be essential for the differentiation, development, and maintenance of professional secretory cells and secretory tissues ^1– 7^. However, UPR functions are not restricted to these tissues, as all key metazoan UPR sensor/transducers are ubiquitously expressed and sustain diverse processes in different tissues ^8– 15^.

ER-resident sensors that detect perturbations of ER composition and function, commonly known as “ER stress”, initiate the UPR (reviewed in 16). ER stress occurs when the folding or degradative capacities of the ER are exceeded (for example, upon expression of mutant proteins that cannot be properly folded or by incapacitation of ER quality-control systems by mutations or pathogens). The UPR transmits information about the ER status to the cell nucleus by inducing the expression of transcription factors ^17– 21^. The UPR also exerts post-transcriptional control by regulating protein synthesis and mRNA stability ^22– 24^. The coordination of transcriptional and post-transcriptional UPR mechanisms determines the cellular decision of whether to adapt or to die if ER stress is unmitigable.

Thousands of articles published to date (a PubMed search for “unfolded protein response” shows more than 7,800 articles, including more than 3,800 in the past five years alone) have revealed the inner workings of the UPR and have portrayed it as a complex network of interconnected signaling pathways. In this review, we discuss new developments in UPR signaling mechanisms, connections among metazoan UPR signaling pathways, and UPR signaling beyond the ER.

## Signal transduction in the UPR: early models and new insights

Three ER–transmembrane stress sensors control the UPR: the kinase/endoribonuclease IRE1, the kinase PERK, and the membrane-tethered transcription factor ATF6 (reviewed in 16). Evolutionary conservation provided the first clues about specialization in UPR signal transduction, which perhaps arose with the advent of multicellularity. IRE1 is ubiquitously found from yeasts to metazoans, whereas PERK and ATF6 appear only in animals ^25^. With regard to ER stress detection, the classic view posits that IRE1, PERK, and ATF6 detect protein-folding perturbations in the ER lumen. Lately, all of them have also been suggested to sense imbalances in lipid composition of the ER membrane ^26– 31^. It has increasingly been recognized that ER deficits leading to proteo- or lipo-toxicity are dealt with by the UPR through different regulatory mechanisms (reviewed in 32). These findings coalesce into a model in which UPR sensors work together holistically by integrating information about distinct/diverse ER deficiencies to launch homeostatic or apoptotic programs.

IRE1 and PERK detect ER stress by direct binding of unfolded protein ligands in the ER lumen, and the reversible dissociation of IRE1 and PERK from the ER lumenal chaperone BiP regulates their activation/deactivation dynamics ^33– 41^. Even though the interaction of BiP with the UPR sensors is long known, other interactions between UPR sensors and ER chaperones have only recently been revealed. For example, the co-chaperone ERDJ4 specifically facilitates BiP’s interaction with IRE1 ^38^, and the activities of both IRE1 and PERK are fine-tuned by various ER chaperones, providing tissue- and context-specific UPR regulation. The protein disulfide isomerase PDIA6 modulates the deactivation of IRE1 and PERK ^42, 43^, and the ER chaperone HSP47 associates with IRE1 but not PERK to regulate its activity ^44^. By contrast, the ER lumenal protein canopy homolog 2 (CNPY2) binds only to PERK during ER stress to regulate downstream signaling ^45^. Moreover, in skeletal muscle cells, the ER oxidoreductase calsequestrin binds IRE1 to inhibit its activity ^46^. These findings support a view wherein the signaling capacity of UPR sensors can be refined to accommodate specific outputs according to the specific needs of cells and tissues. Most likely, the aforementioned interactions of UPR sensors with chaperones also influence the temporal dynamics of UPR signaling activation and deactivation, thereby providing an additional level of regulatory control leading to the life versus death decision in cells ^47– 50^. In this way, modulation of the UPR at the level of UPR sensor–chaperone interactions serves as an initial point for information integration to regulate complex downstream UPR outputs.

How ATF6, by comparison with IRE1 and PERK, detects ER stress is less well understood. The established model posits that ER stress leads to ATF6 export to the Golgi apparatus, where resident proteases cleave it to liberate a soluble transcription factor, ATF6N ^51, 52^. Accumulating evidence suggests that ATF6 may act as a redox sensor or that it is coupled to one, as the reduction of intra- and inter-molecular disulfide bonds in its ER lumenal domain appears to be required for trafficking to the Golgi apparatus ^53^. The recent observation of aberrant and attenuated ATF6 signaling in cells deficient in the oxidoreductase ERp18, which associates with ATF6 during ER stress, supports this view ^54^.

Self-association/dissociation of UPR sensors is another canonical feature of the UPR. ER stress–induced oligomerization of IRE1 and PERK in the plane of the ER membrane drives the *trans*-autophosphorylation of their respective cytosolic kinase domains (reviewed in 16). Active PERK phosphorylates eIF2α (the alpha subunit the eukaryotic translation initiation factor 2), causing a decrease in global protein synthesis ^24^. Paradoxically, a subset of mRNAs bearing small upstream open reading frames (uORFs) on their 5′ untranslated regions (5′ UTRs) are preferentially translated in these conditions, including those encoding the transcription factors ATF4 and CHOP ^21, 55– 58^. By increasing the cellular metabolic capacity, ATF4 effects are mostly cytoprotective ^59^ whereas CHOP is largely regarded as pro-apoptotic ^60– 63^. It has recently been shown that, in addition to translational control by uORFs, N6-methyladenosine (m6A) methylation of the *ATF4* mRNA also contributes to the control of its translation. Demethylation of the 5′ UTR of the *ATF4* mRNA by the demethylase ALKBH5 enhances its translational re-initiation, promoting the synthesis of ATF4 during ER stress ^64^. This mechanism highlights the exquisite fine-tuning of protein synthesis re-programing during stress.

Unlike PERK, IRE1 has no known kinase substrates besides itself, and its active kinase domain licenses the allosteric activation of its C-terminal RNase domain ^65, 66^. Active IRE1 excises a small unconventional intron from the mRNA encoding the transcription factor XBP1 ^18, 19^. The resulting exons are joined by the tRNA ligase RTCB to produce a new mRNA encoding the transcription factor XBP1S (“S”, for spliced) ^67– 69^. Even though *XBP1* mRNA splicing was discovered almost 20 years ago, some of the salient features of this mechanism have been elucidated recently. Five years ago, RTCB was independently identified by three groups as the *XBP1* mRNA splicing ligase ^67– 69^. A conformational change in the *XBP1* mRNA, dubbed an RNA “zipper”, which is required to eject the intron and hold the exons together after cleavage, was described shortly after ^70^. Additional recent work has shown that an intact 2′-3′ cyclic phosphate—long known to be left on the RNA ends after cleavage by IRE1 ^71^—is essential for completion of the *XBP1* mRNA splicing reaction ^72^. The opposing activities of the cyclic phosphodiesterase CNP and the RNA cyclase RTCA control the availability of the cyclic phosphate ^72^. Targeted quantitative proteomics analyses revealed that IRE1 is found in complex with RTCB in cells ^73^, suggesting that the *XBP1* mRNA splicing can be completed immediately after mRNA cleavage. This newly described multi-step regulation of *XBP1* mRNA splicing could also provide regulatory layers controlling a tunable UPR output.

IRE1 signaling is not confined to *XBP1* mRNA splicing. IRE1 also cleaves ER-bound mRNAs in a process known as regulated IRE1-dependent decay (RIDD) ^22, 23^. When first discovered, RIDD was thought to protect the ER by lowering ER load through the selective cleavage of mRNAs ^23^; however, a recent finding challenges this view. RIDD of a single mRNA encoding the lysosome trafficking factor BLOS1 has been shown to protect cells from proteotoxicity by enhancing their capacity to degrade protein aggregates by microautophagy ^74^. The precise molecular mechanism that determines the fate of an mRNA encountering IRE1—splicing or RIDD—appears to hinge on the aforementioned *XBP1* mRNA zipper, which is absent in RIDD targets studied to date; implanting this mRNA zipper structure into RIDD target mRNAs results in their splicing ^75^.

Our understanding of IRE1 signaling mechanisms has also expanded lately. Several lines of evidence support the notion that IRE1 is an integrating node linking the UPR and the ER protein co-translational targeting machinery. IRE1 has been shown to bind to the Sec61 translocon ^76^, and impairing this interaction resulted in dysregulated IRE1 activity ^77^. The recently developed Perturb-seq method, which combines single-cell RNA-seq with CRISPR-based genetic screens, further substantiated these observations by showing that depletion of translocon subunits resulted in exclusive IRE1 activation without impact on other UPR signaling pathways ^78^. More recently, RNA–protein cross-linking and mass spectrometry–based approaches revealed that IRE1 associates with the signal recognition particle, tRNAs, mRNAs, and ribosomes in living cells ^73^. All of these observations converge on a model in which IRE1 oversees the health and availability of translocons while it monitors the co-translational targeting machinery at the ER surface.

## Cross-talk between UPR signaling pathways controls adaptation and death

A basic level of pathway interconnectivity in the UPR comprises the coordinated actions of transcription factors. In the adaptive phase of the UPR, ATF6N and XBP1S increase the synthesis of chaperones, protein-folding enzymes, and proteins that take part in ER protein turnover mechanisms, and they physically enlarge the ER by upregulating endomembrane biosynthesis ^9, 79– 83^. In parallel, ATF4 upregulates the biosynthetic capacity of the cell by controlling genes required for antioxidant responses and amino acid import ^59^. Adaptive transcriptional signals further integrate at the level of combinatorial regulation. For example, ATF6N and XBP1S can form heterodimers ^84^, thereby expanding the repertoire of UPR *cis*-regulatory elements ^9, 20, 83, 85, 86^. Post-transcriptional regulatory control by XBP1U (“U” for unspliced), encoded by the *XBP1* precursor mRNA, adjusts the transcriptional responses; XBP1U regulates the turnover of XBP1S and ATF6N, setting a molecular timer for the duration of the adaptive phase of the UPR ^87, 88^.

Not all UPR transcriptional outputs are adaptive. ATF4, ATF6N, and XBP1S converge on the induction of CHOP ^9, 21, 83, 85^, which negatively impacts the ER protein-folding capacity, downregulates the anti-apoptotic protein BCL2, and upregulates the pro-apoptotic signaling protein death receptor 5 (DR5) ^50, 61, 63, 89, 90^. However, recent data showing that genetic depletion of ATF6—but not IRE1 or PERK—significantly impaired the growth of HeLa cells engineered to adopt a plasma cell–like secretory phenotype that predisposes them to ER stress suggest that ATF6 is mostly cytoprotective ^91^. The homeostatic role of the IRE1–XBP1 axis has recently been challenged by the discovery of cytotoxic XBP1-driven responses ^92^. These data show that surpassing a critical ER stress threshold allows XBP1S to indirectly induce the expression of the ER calcium channels TMEM38B and ITPR1, leading to depletion of ER calcium stores, thereby providing a positive feedback loop to aggravate ER stress ^92^. In this model, cellular demise arises from collateral damage caused by the transcription factor KLF9 downstream of XBP1S and not by the direct control of *bona fide* pro-apoptotic genes by XBP1S.

XBP1-independent roles with regard to cytoprotection or apoptosis have also been proposed for IRE1. One model suggests that IRE1 promotes cell death by degrading microRNAs (miRNAs) targeting mRNAs encoding the pro-apoptotic proteins TXNIP and caspase-2 ^93, 94^. This model is somewhat disputed, as caspase-2 appears to be dispensable for eliciting cell death in response to ER stress ^50, 95^. Another view posits that RIDD could lead to cell death through pervasive RNA cleavage ^96^. The notion of antagonistic IRE1 roles has been further substantiated by a recent finding showing that XBP1S promotes glioblastoma tumor progression while RIDD obstructs it ^97^.

Antagonism among UPR sensors also provides a model for UPR-dependent cell fate control. This model considers a dynamic cross-talk between cytoprotective and pro-apoptotic signals emanating from different UPR sensors. IRE1 promotes cytoprotection through *XBP1* mRNA splicing and RIDD, while PERK promotes apoptosis by inducing the ATF4–CHOP axis and the gene encoding DR5 downstream of it ^50^. DR5 signaling instructs the cells to die in response to unmitigable ER stress through the extrinsic (caspase-8–dependent) apoptotic pathway; IRE1 combats the apoptotic signal by degrading the *DR5* mRNA ^50^. Concomitantly, the phosphatase RPAP2, downstream of PERK, dephosphorylates IRE1 attenuating IRE1 signaling ^98^. Along with RPAP2, caspase-mediated IRE1 turnover can also play a role in terminating signaling and enforcing the apoptotic program ^99^. Surprisingly, cleavage of IRE1 by caspases also generates a proteolytic fragment that antagonizes BAX-driven pro-apoptotic signaling from the mitochondria ^99^, further substantiating the notion of extensive functional cross-talk between the UPR and other fundamental cellular processes.

PERK also stimulates the production of GADD34, a regulatory subunit of PP1 (protein phosphatase 1), which dephosphorylates eIF2α, thereby establishing a negative feedback loop controlling responses downstream of PERK ^100, 101^. The cross-connectivity between UPR signaling pathways has been further substantiated by recent RNA-seq and ribosome profiling experiments that show that PERK has the potential to repress—both transcriptionally and post-transcriptionally—the expression of a subset of cytoprotective genes induced by XBP1S and ATF6N ^102^. Together, these observations support a model in which a molecular timer, set off by opposing signals downstream of IRE1 and PERK, coordinates the survival versus death decision. In this model, the activities of IRE1 and PERK are coordinated, ensuring that specific responses kick in as the response to ER stress progresses. At the beginning of the response, homeostatic mechanisms are enforced while apoptotic ones are suppressed (for example, induction of *XBP1* mRNA splicing, RIDD of the *DR5* mRNA). If stress persists, apoptosis mechanisms take over (for example, shutdown of IRE1 signaling, suppression of cytoprotective genes downstream of XBP1S and ATF6).

Apart from the mechanisms discussed above, protein quality-control mechanisms of UPR sensors have been implicated in UPR signaling; the protein levels of IRE1, PERK, and ATF6 are controlled by ER-associated degradation (ERAD) ^103– 105^. These observations suggest that ERAD engages negative feedback loops that could enforce the survival versus death decision. Moreover, recent data suggest that post-translational modification of IRE1 and PERK by ufmylation, which regulates their stability, plays an important role in regulating apoptosis and plasma cell development ^106, 107^. Importantly, post-transcriptional mechanisms operate alongside protein turnover devices to regulate the UPR. It has increasingly been recognized that non-coding RNAs—mostly miRNAs but also long non-coding RNAs—act as important regulators of the UPR (reviewed in 108). This type of regulatory control adds a layer of complexity to the homeostatic or apoptotic programs controlled by the UPR.

## Beyond the ER: non-canonical UPR mechanisms and interorganellar communication

As we move forward in our understanding of the UPR, we have come to recognize that UPR signaling extends beyond protecting ER physiology. Recent observations support an integral role of the UPR as a hub for interorganelle communication. At ER–mitochondria membrane contact sites, IRE1 coordinates mitochondrial physiological bioenergetics by engaging the calcium channel ITPR ^109^. IRE1 regulation at ER–mitochondria junctions also tunes biomedically relevant processes, such as the regulation of T-cell responses ^110^. In parallel, PERK stimulates the assembly of respiratory chain supercomplexes in a nutritional and ER stress–dependent manner ^111, 112^. The UPR–mitochondria interplay is bidirectional: a genomic high-content screen in yeast demonstrated that mitochondrial heme biosynthesis enables optimal UPR signaling ^113^. More recently, the mitochondrial ubiquitin ligase MITOL was shown to ubiquitylate IRE1 at ER–mitochondria contact sites to suppress its activity and prevent apoptosis ^114^. These studies showcase the conserved role of the UPR as an integration node for interorganelle communication.

IRE1 and PERK are also found at ER–plasma membrane (ER-PM) contact sites through their association with the cytoskeletal scaffold filamin A (FLNA). PERK-driven FLNA recruitment expands ER-PM contacts and replenishes ER calcium stores ^115^, while IRE1 engages FLNA to facilitate cell motility ^116^. These UPR mechanisms are independent of the enzymatic activities of IRE1 or PERK, relying instead on dynamic UPR sensor clustering. Unexpectedly, the role of UPR–organelle cross-talk in modulating UPR signaling was further illustrated by the observation that ceapins, recently discovered ATF6 signaling inhibitors ^117, 118^, work by providing a neomorphic artificial tether between the ER and peroxisomes ^119^.

In spite of the compelling evidence supporting non-canonical roles for IRE1 signaling, a recent report demonstrated that *XBP1* mRNA splicing is the only IRE1 activity required for medaka fish development and growth ^120^. However, this observation does not negate the relevance of cross-connectivity between the UPR and other cellular processes in maintaining organismal homeostasis. Far from behaving as a self-contained transcriptional program, the UPR drives the expression of distinct sets of genes in a cell type- and stimulus-dependent manner that involves metabolic, inflammatory, or developmental cues ^9, 121, 122^. Moreover, out of hundreds of UPR regulated genes, many have no direct functions in maintaining ER homeostasis. For example, some UPR target genes coordinate the DNA damage and repair response, hinting at a key role for UPR in maintaining genome integrity ^9, 122, 123^. Together, canonical and non-canonical mechanisms emerging from different UPR sensors—at least for IRE1 and PERK—assemble into a multipronged signaling relay that most likely determines the robustness of the UPR while coordinating the activities of other organelles and functions within the cell.

## UPR signal integration at the organism level

The interconnected nature of UPR signaling is not restricted to single cells, as it has been increasingly clear that a cell non-autonomous UPR plays a role in maintaining organismal health. Work in *Caenorhabditis elegans* showed that IRE1 regulates stress resistance and longevity through an unidentified diffusible factor, which is a downstream target of XBP1 ^124^. Moreover, ectopic expression of XBP1S in mouse neurons leads to stress responses in liver and improves hepatic insulin sensitivity ^125^. More recently, a role for UPR in the maintenance (or disruption) of circadian rhythms in animals has been elucidated. Through the combined action of transcriptional and translational mechanisms, the UPR regulates the expression of circadian factors to ensure the oscillatory control of metabolic rhythms ^126, 127^. On the flip side, under ER stress conditions, PERK inhibits the circadian heterodimeric components BMAL1 and CLOCK, abrogating cellular rhythms in cells and animals ^128, 129^.

The fundamental roles of the UPR in coordinating organism-level homeostasis are further accentuated by the potential for therapeutic utility of recently discovered small-molecule modulators of the UPR. In some instances, it may be desirable to suppress UPR signaling for a favorable outcome, as has been recently demonstrated for pharmacologic inhibition of IRE1 in orthotopic models of multiple myeloma ^130^ and atherosclerosis ^131^ or by blocking IRE1 functions as a promising strategy for pain management ^132^. In other instances, it may be desirable to enhance UPR signaling to increase the protein processing capacity of the ER. Recently discovered selective activators of ATF6 show promise in this regard as they alleviate amyloidogenic protein aggregation and proteotoxicity in various disease models, including myocardial ischemia ^133– 135^. The positive effects of the small-molecule ISRIB, which renders cells insensitive to the effects of eIF2α phosphorylation ^136^, on models of neurological disorders and brain injury further substantiate this notion. For example, ISRIB rescues the effects of genetic mutations observed in vanishing white matter disease ^137^, reverses cognitive deficits in mouse models of traumatic brain injury ^138^, alleviates social behavioral defects and anxiety-like symptoms in a genetic mouse model ^139^, and protects neural cells in mouse models of prion disease ^140^. Importantly, a small molecule called Sephin1, which has been proposed to obstruct eIF2α dephosphorylation, also provides therapeutic benefit in mouse models of protein misfolding neuropathologies ^141– 143^. These findings highlight the potential for controlling eIF2α phosphorylation in different pathologies. All of these observations underscore the notion that the pharmacological manipulation of the UPR is an attractive opportunity for targeted therapeutic intervention in multiple diseases.

Taken together, the aforementioned findings portray a bottom-up view of the UPR homeostat that extends beyond the scope of maintaining ER homeostasis. In the UPR hierarchy, at its most basic level, the UPR maintains ER physiology. In the next level, the UPR interdigitates with cellular physiology through dynamic connections between the ER and other cellular components and processes. In a higher plane, the UPR integrates signals at the multicellular level, facilitating communication about specific states between cells and tissues and modulating the oscillatory dynamics of organismal rhythms. By interlocking fundamental biological processes at multiple levels, the UPR emerges as a main regulatory hub that maintains the health of the whole organism.

## What does the future hold for the UPR signaling mechanisms?

The past five years have seen great progress in our understanding of the UPR. These developments raise new questions. For example, although we know that clustering is essential for UPR sensor activation, we do not understand the mechanisms that would drive their spatiotemporal organization in specific domains of the ER membrane. We also know very little about the mechanistic details that underlie the functional partitioning of each UPR signaling pathway. Is the main function of IRE1 to survey translocon integrity and availability while other homeostatic functions are delegated to ATF6? Are pro-apoptotic functions limited mostly to PERK? The significance of other molecular mechanisms also remains obscure. Are there other RIDD substrates that, akin to BLOS1, control specific cytoprotective or pro-apoptotic responses? Is the ribosome a signaling hub for IRE1 or PERK? Do non-canonical UPR mechanisms impact the differentiation of cells and tissues, or do they exist simply to fine-tune cellular responses to specific physiological states? These questions will provide fertile ground for research for years to come.

Another recent observation that begets new questions is the finding that not every cell in a population responds in the same way to the same ER stress input ^78^. Is it possible that, akin to the innate antiviral type-I interferon response, alerting neighboring cells about a potential threat to ER functions allows coordinated responses that maintain tissue and organism homeostasis? Is it possible that cells exhibit different UPR types depending on whether they are actively cycling, have exited the cell cycle irreversibly in preparation for terminal differentiation, or are terminally differentiated? Future work addressing these questions could reveal new UPR signaling paradigms in multicellular organisms.

The differences in the response to ER stress of individual cells in an asynchronous population strongly hint at a fundamental connection between UPR signaling and the cell cycle. Substantiating this notion, the yeast XBP1 homolog Hac1 has been implicated in cytokinesis ^144^, and blocking IRE1 signaling delayed cell cycle progression in helper T-cells ^145^. In addition, PERK signaling has been proposed to negatively impact cell cycle progression ^146, 147^. A putative role of XBP1U in controlling cell proliferation has recently been discovered: XBP1U acts as a negative regulator of the p53/p21 tumor suppressor axis, revealing a potential oncogenic role for this protein ^148^. Even though this role is independent of IRE1, it is possible that it remains linked to the UPR since both ATF6N and XBP1S induce *XBP1* mRNA expression ^9, 19^. Future work is required to uncover the mechanistic details behind a putative “ER health checkpoint” in the metazoan cell cycle.

The developments discussed here highlight that the UPR story is far from complete. They portray a far more complex molecular circuitry for the UPR than previously anticipated. This new knowledge on basic UPR mechanisms has the potential to dramatically change our outlook on how to manipulate the UPR for therapeutic intervention. It is becoming clear that the UPR is much more than the sum of its parts and that this Rube Goldberg stress signaling device will continue to gift significant discoveries for years to come.

## Abbreviations

5′ UTR, 5′ untranslated region; ALKBH5, AlkB homolog 5 RNA demethylase; ATF4, activating transcription factor 4; ATF6, activating transcription factor 6; BAX, BCL2-associated X protein; BCL2, apoptosis regulator BCL2/B-cell CLL/lymphoma 2; BiP, binding-immunoglobulin protein; BLOS1, biogenesis of lysosome-related organelles complex 1 subunit 1; BMAL1, brain and muscle ARNT-like 1; CHOP, CCAAT/enhancer-binding protein homologous protein; CLOCK, circadian locomoter output cycles protein kaput; CNP, 2′,3′ cyclic nucleotide 3′ phosphodiesterase; CNPY2, protein canopy homolog 2; CRISPR, clustered regularly interspaced short palindromic repeats; DR5, death receptor 5; eIF2α, eukaryotic translation initiation factor 2, subunit alpha; ER, endoplasmic reticulum; ERAD, ER-associated degradation; ERDJ4, ER DnaJ homolog 4; ERp18, endoplasmic reticulum resident protein of 18 kDa; FLNA, filamin A; GADD34, growth arrest and DNA damage-inducible 34/PP1 regulatory subunit 15A; HSP47, heat shock protein 47; IRE1, inositol-requiring enzyme 1; ISRIB, integrated stress response inhibitor; ITPR1, inositol 1,4,5-trisphosphate receptor type 1; KLF9, Krueppel-like factor 9; m6A, N6-methyladenosine; miRNA, microRNA; MITOL, mitochondrial ubiquitin ligase; PDIA6, protein disulfide isomerase family A member 6; PERK, PKR-like endoplasmic reticulum kinase; PM, plasma membrane; PP1, protein phosphatase 1; RIDD, regulated IRE1-dependent decay; RPAP2, RNA polymerase II-associated protein 2; RTCA, RNA 3′-terminal phosphate cyclase; RTCB, tRNA-splicing ligase RtcB homolog; TMEM38B, transmembrane protein 38B; TXNIP, thioredoxin-interacting protein; uORF, upstream open reading frame; UPR, unfolded protein response; XBP1, X-box binding protein
